# *Burkholderia pseudomallei* modulates host iron homeostasis to facilitate iron availability and intracellular survival

**DOI:** 10.1371/journal.pntd.0006096

**Published:** 2018-01-12

**Authors:** Imke H. E. Schmidt, Claudia Gildhorn, Martha A. L. Böning, Vera A. Kulow, Ivo Steinmetz, Antje Bast

**Affiliations:** 1 Friedrich Loeffler Institute of Medical Microbiology, University Medicine Greifswald, Greifswald, Germany; 2 Institute of Hygiene, Microbiology and Environmental Medicine, Medical University of Graz, Graz, Austria; University of Tennessee, UNITED STATES

## Abstract

**Background:**

The control over iron homeostasis is critical in host-pathogen-interaction. Iron plays not only multiple roles for bacterial growth and pathogenicity, but also for modulation of innate immune responses. Hepcidin is a key regulator of host iron metabolism triggering degradation of the iron exporter ferroportin. Although iron overload in humans is known to increase susceptibility to *Burkholderia pseudomallei*, it is unclear how the pathogen competes with the host for the metal during infection. This study aimed to investigate whether *B*. *pseudomallei*, the causative agent of melioidosis, modulates iron balance and how regulation of host cell iron content affects intracellular bacterial proliferation.

**Principal findings:**

Upon infection of primary macrophages with *B*. *pseudomallei*, expression of ferroportin was downregulated resulting in higher iron availability within macrophages. Exogenous modification of iron export function by hepcidin or iron supplementation by ferric ammonium citrate led to increased intracellular iron pool stimulating *B*. *pseudomallei* growth, whereas the iron chelator deferoxamine reduced bacterial survival. Iron-loaded macrophages exhibited a lower expression of NADPH oxidase, iNOS, lipocalin 2, cytokines and activation of caspase-1. Infection of mice with the pathogen caused a diminished hepatic ferroportin expression, higher iron retention in the liver and lower iron levels in the serum (hypoferremia). *In vivo* administration of ferric ammonium citrate tended to promote the bacterial growth and inflammatory response, whereas limitation of iron availability significantly ameliorated bacterial clearance, attenuated serum cytokine levels and improved survival of infected mice.

**Conclusions:**

Our data indicate that modulation of the cellular iron balance is likely to be a strategy of *B*. *pseudomallei* to improve iron acquisition and to restrict antibacterial immune effector mechanisms and thereby to promote its intracellular growth. Moreover, we provide evidence that changes in host iron homeostasis can influence susceptibility to melioidosis, and suggest that iron chelating drugs might be an additional therapeutic option.

## Author summary

Iron is an essential nutrient for many bacterial pathogens. A sufficient availability is linked to bacterial proliferation and pathogenicity. The host requires iron for cellular functions including innate immune defense mechanisms. Consequently, the control over iron homeostasis plays an important role in the course of infection. *Burkholderia pseudomallei* is an environmental bacterium ubiquitous in soil and water surfaces causing the disease melioidosis with a wide range of signs and symptoms including localized, pulmonary, or bloodstream infections. Conditions with increased iron stores, such as thalassemia, are considered to increase the risk to acquire melioidosis. Here we show that infection with the pathogen triggers downregulation of the major cellular iron exporter inducing intracellular iron retention and stimulation of bacterial proliferation. Experimental iron overload appears to predispose to infection with *B*. *pseudomallei*, whereas iron deficiency confers relative resistance to melioidosis. These effects of changed iron metabolism on the course of infection may be ascribed to modifications in the host immune response and direct effects on bacterial growth, respectively. Thus, the *B*. *pseudomallei*-driven alteration of cellular iron traffic leading to increased iron availability can promote its intracellular growth, and treatment with iron chelators together with antibiotics might be an appropriate strategy to control infection.

## Introduction

Iron plays a central role at the host-pathogen interface, because it is a pivotal nutrient for microbial pathogens and their mammalian hosts. Both require the metal as a cofactor or as prosthetic group for key enzymes involved in fundamental cellular functions and metabolic processes [1]. Iron affects microbial growth and pathogenicity but also impacts on immune cell plasticity and responses [2].

Macrophages are essential for immune function and also contribute to maintenance of iron homeostasis. They acquire heme iron by phagocytosis of senescent or damaged erythrocytes and receptor-mediated uptake of proteins binding extracellular free heme (via interaction of hemopexin with CD91) or haemoglobin (via interaction of haptoglobin with CD163). The heme porphyrin ring is subsequently cleaved by heme oxygenase-1 (HO-1) to release carbon monoxide (CO), biliverdin and ferrous iron. Apart from erythrophagocytosis macrophages can also obtain transferrin-bound iron by transferrin receptor (TfR1, CD71)-mediated endocytosis or non-transferrin-bound iron via divalent metal transporter 1 (Dmt1, Nramp2, Slc11a2). The natural resistance macrophage protein 1 (Nramp1, Slc11a1) is able to pump the metal from late endosomes and phagolysosomes into the cytoplasm. Macrophages also use the lipocalin 2 receptor to acquire both iron-laden and iron-free complexes of siderophores and lipocalin 2 (Lcn2, NGAL, 24p3, siderocalin) [3]. Excess supply of intracellular iron is stored in ferritin to avoid toxic effects of free iron, which carries pro-oxidative properties [2] or can be exported by the transmembrane protein ferroportin (Fpn, Slc40a1). The expression of both proteins is increased by heme and iron [4, 5].

The export of iron from macrophages is controlled systemically by hepcidin, an acute-phase peptide, secreted by hepatocytes and to a minor extent by macrophages. Hepcidin binds to ferroportin, mainly expressed on the surface of macrophages and duodenal enterocytes, and triggers its internalization and proteasomal degradation. This results in sustained inhibition of cellular iron efflux, leading to intracellular iron accumulation and a drop in serum iron levels (hypoferremia). The expression of the hepcidin antimicrobial peptide (Hamp) is regulated primarily at the transcription level, being induced by hyperferremia, cytokines including IL-1 and IL-6, or recognition of lipopolysaccharide, and repressed by hypoferremia, anemia or hypoxia [6].

Limitation of iron availability from invading pathogens represents a key pathway in host defense, a process named nutritional immunity. The general strategy to restrict iron access to extracellular pathogens consists in the retention of intracellular iron by macrophages leading to hypoferremia. This is achieved essentially by the induction of iron import and retention mechanisms, coupled to suppression of cellular iron export. In contrast, the main strategy to limit iron availability to intracellular pathogens involves hyperferremia, driven by systemic induction of cellular iron export together with the suppression of cellular iron import systems from macrophages [6]. Thus, successful pathogens must employ mechanisms to evade nutritional immunity to cause disease [7].

*Burkholderia pseudomallei* is a gram-negative, non-spore forming bacterium which is found world-wide in tropical and subtropical areas with its major endemic regions in Southeast Asia and northern Australia. Its natural habitat is moist soil or surface water, from which humans acquire the often fatal disease melioidosis with a huge variety of clinical manifestations including pneumonia, abscess formation or septicaemia following bacterial inoculation, inhalation, or ingestion. *B*. *pseudomallei* is able to enter, survive and replicate within mammalian host cells by the use of a type three secretion system [8]. However, limited data are available about the role of important nutrients such as iron to the pathogenesis of *B*. *pseudomallei*.

Previous work has pointed toward a link between high iron levels and the occurrence of the bacterium in soil or water samples [9–12]. Iron supplementation in soil can affect the growth of *B*. *pseudomallei* [13], changes the pathogen’s morphology from a rod-like to coccoid form, which may be advantageous for its persistence in the environment and may rise the risk of its transmission to humans [14] and increases biofilm formation as well [15].

*B*. *pseudomallei* produces a hydroxamate-type siderophore, malleobactin that is able to capture iron from host proteins transferrin and lactoferrin, enabling the pathogen to grow under iron-deficient conditions [16–18]. Other studies have identified several genes encoding iron acquisition systems such as for the siderophore pyochelin, the uptake of heme or plasma membrane iron transporters [19–22]. Using mutants deficient in siderophore and/or heme utilization systems, Kvitko et al. [23] found that the use of lactoferrin was reliant on malleobactin (*mba*), but not pyochelin (*pch*) synthesis and/or uptake, and that acquisition of hemin/hemoglobin was only reliant on the *hmu*, but not the *hem* locus. Surprisingly, a quadruple mutant remained completely virulent in a murine model and was able to grow in the presence of ferritin that might serve as an alternate iron source. *B*. *pseudomallei* has not only the ability to grow under iron limited conditions but further studies revealed that conditions with enhanced iron stores such as thalassemia or idiopathic pulmonary hemosiderosis are risk factors for melioidosis [24–31].

We have previously shown that upregulation of HO-1 expression occurs during *B*. *pseudomallei* infection and both HO-1 and HO-1-derived CO favor the establishment of the disease [32]. However, due to limited information about the contribution of the HO-1 metabolite iron during *B*. *pseudomallei* infection, we aimed to define the changes of macrophage iron homeostasis upon infection and to elucidate the importance of such modifications for host susceptibility or resistance to infection. We found that *B*. *pseudomallei* has developed strategies to circumvent iron restriction mechanisms of the host and that alterations in iron homeostasis can influence the course of murine melioidosis.

## Materials and methods

### Ethics statement

Animal experiments were carried out in agreement with the recommendations in the Guide for the Care and Use of Laboratory Animals of the National Institutes of Health. All studies were approved by the Landesamt für Landwirtschaft, Lebensmittelsicherheit und Fischerei Mecklenburg-Vorpommern (LALLF M-V; 7221.3–1.1-020/11; 7221.3-1-028/14).

### Mice

C57BL/6J mice were obtained from The Jackson Laboratory (Bar Harbor, Maine, US). Breeding was done by MICROMUN (Greifswald, Germany) and the Department of Laboratory Animal Science of the University Medicine of Greifswald (Greifswald, Germany), respectively. Mice (female, 10–12 weeks) were kept in filter-top cages under standard laboratory conditions (biosafety level 3) with free access to food and water.

### Bacteria

*B*. *pseudomallei* strain E8 is a soil isolate from Northeast Thailand [33] and was used during this study. Bacteria were grown on Columbia agar with 5% sheep blood (BD Biosciences, Heidelberg, Germany) at 37°C for 24 hours and adjusted to the desired concentration in Dulbecco’s phosphate-buffered saline (D-PBS; Life Technologies, Darmstadt, Germany) or cell culture medium. For experiments using heat-inactivated *B*. *pseudomallei*, bacteria were suspended in D-PBS, incubated at 70°C for 20 min and stored at -70°C until use.

### Bacterial growth

Bacteria were grown on blood agar at 37°C for 24 hours, adjusted to an optical density (OD) at 650 nm of 0.01 or 0.05 in 15 ml of Lennox LB broth or iron-free M9 minimal medium, and incubated with or without the ferric ammonium citrate (FAC, 100 μM, Sigma- Aldrich, Taufkirchen, Germany), deferoxamine mesylate salt (DFO, 50 μM, Sigma-Aldrich), hepcidin-1 trifluoroacetate salt (1 μg/ml, Bachem, Bubendorf, Switzerland), or the corresponding vehicle (A. bidest) at 37°C with shaking at 140 rpm. In accordance with the time points used for the antibiotic protection and lactate dehydrogenase assay, the OD_650_ of bacterial cultures were measured at 0, 6, and 24 hours. Bacterial growth was determined as described [32].

### Culture of Hepa1-6 cells

Murine hepatoma Hepa1-6 cells (ACC 175) were obtained from the Leibnitz Institute DSMZ (German Collection of Microorganisms and Cell Cultures, Braunschweig, Germany). Cells were cultured in Dulbecco’s MEM with 4.5 g/l glucose (Life Technologies) complemented with 10% heat-inactivated fetal calf serum (PAN Biotech, Aidenbach, Germany) at 37°C in a humidified atmosphere containing 95% air and 5% CO_2_.

### Generation and cultivation of primary murine macrophages

Bone marrow-derived macrophages (BMM) were generated and cultivated in a serum-free cell culture system as recently described [34].

### Antibiotic protection assay

20 hours prior to infection, BMM (1.5 x 10^5^ cells per well) or Hepa1-6 cells (6 x 10^4^ cells per well) were seeded in 48 well plates, and where applicable treated with FAC, DFO, hepcidin or the corresponding vehicle. As described in our recent study [32], BMM were infected with *B*. *pseudomallei* strain E8 at the indicated multiplicity of infection (MOI) and centrifuged. After infection for 30 min, cells were washed and incubated in kanamycin (BMM: 100 μg/ml; Hepa1-6 cells: 250 μg/ml)-containing medium (with additives). At defined time points (time zero was taken 30 min after incubation with antibiotic-containing medium), the number of intracellular colony forming units (CFU) was determined by plating serial dilutions of lysed cells on LB agar.

### Lactate dehydrogenase assay

To quantify the extent of membrane-damaged cells in response to bacterial infection, we measured the release of lactate dehydrogenase (LDH) in cell culture supernatants as described [32].

### RNA isolation and quantitative real-time PCR (qRT-PCR)

20 hours prior to infection, BMM (6.5 x 10^5^ cells per well) were seeded in 6 well plates, and where applicable treated with FAC, DFO, hepcidin or the corresponding vehicle (A. bidest). BMM were infected with *B*. *pseudomallei* strain E8 at the indicated MOI and centrifuged.

RNA isolation and qRT-PCR were performed as described [32]. Data were analysed by advanced relative quantification (efficiency method) with LightCycler software version 1.5. The relative expression ratio of a target gene was computed, based on its real-time PCR efficiencies (E), and the crossing point (CP) difference (Δ) of one unknown sample versus one control. The expression of the target gene was normalized by the expression of the non-regulated reference gene RPLP0 (ribosomal protein large P0) relative to a calibrator sample. All assays were done in duplicate and repeated as indicated in the figure legends.

### Protein extraction and western blot analysis

Protein preparation, SDS-PAGE and western blot analysis were conducted as described [35]. Rabbit anti-GAPDH (AbFrontier, Acris Antibodies, Herford, Germany), rabbit anti-heme oxygenase-1 (HO-1; Enzo Life Sciences, Lörrach, Germany), rabbit anti-caspase-7 (Cell Signaling Technology, New England Biolabs GmbH) antibody (in 20 mM Tris, 138 mM NaCl, pH 7.6, 5% (w/v) BSA, 0.1% (v/v) Tween^®^ 20), or rabbit anti-caspase-1 (Santa Cruz Biotechnology, Heidelberg, Germany, in 1x Roti^®^-Block), and horseradish peroxidase (HRP)-conjugated anti-rabbit IgG (in 1x Roti^®^-Block, Cell Signaling Technology) were used as primary and secondary antibodies, respectively.

### Immunofluorescence staining

20 hours prior to infection, BMM (2 x 10^5^ cells per well) were seeded on coverslips in 24 well plates, infected with *B*. *pseudomallei* strain E8 at the indicated MOI and centrifuged. 24 hours after infection cells were washed with ice-cold D-PBS. Fixation and blocking steps were done as described [32]. Polyclonal rabbit anti-ferroportin (Thermo Fisher Scientific (Life Technologies), Darmstadt, Germany) or monoclonal mouse anti-*B*. *pseudomallei* 3015γ2b antibody [36] (both in IF buffer), and red fluorescent Cy3-conjugated goat anti-rabbit IgG or green fluorescent Alexa Fluor 488 anti-mouse IgG2b (in IF buffer) were used as primary and secondary antibodies, respectively. Slices were observed by fluorescence microscopy with a BZ-9000 microscope (Keyence, Neu-Isenburg, Germany).

### Measurement of intracellular labile iron

20 hours prior to infection, BMM (1.5 x 10^5^ cells per well) were seeded in 48 well plates, and where appropriate treated with FAC, DFO, hepcidin or the corresponding vehicle (A. bidest), followed by infection with *B*. *pseudomallei* strain E8 at MOI 50. Well plates were centrifuged for 4 min at 120 x g. 24 hours after infection, BMM were washed twice with Hank’s Balanced Salt Solution (HBSS; Sigma-Aldrich). After staining with 25 μM of the green-fluorescent Fe^2+^ indicator Phen Green SK, diacetate (Thermo Fisher Scientific), BMM were incubated for 30 min at 37°C and 5% CO_2_. 10 min before the end of the incubation period, the blue-fluorescent nuclear staining dye Hoechst (1 μg/ml) was added. After three washing steps with HBSS, the fluorescence intensity was measured at an excitation wavelength of 507 nm and emission wavelength of 532 nm for Phen Green SK, and at an excitation wavelength of 350 nm and emission wavelength of 461 nm for Hoechst using the microplate reader Infinite M200 PRO (Tecan). The ratio of Phen Green SK and Hoechst was calculated, the relative fluorescence was normalized to the mean of the untreated and uninfected control, which was set one, and used for determination of intracellular iron content. Upon binding iron Phen Green SK fluorescence is quenched and is therefore inversely correlated with intracellular iron content. Thus, a decrease of the relative fluorescence is indicative for an increase of iron in BMM.

### *In vivo* infection

Experimental melioidosis was induced by intranasal inoculation of mice with *B*. *pseudomallei* as described by Stolt et al. [32]. Data are presented as the total bacterial count (CFU) per organ. FAC (5 mg/kg), DFO (100 mg/kg) or vehicle (D-PBS) were administered intraperitoneally 30 minutes before *B*. *pseudomallei* inoculation and 24 hours after infection and continued until sacrifice.

### Serum and bronchoalveolar lavage fluid (BALF)

Blood and BALF were obtained as described [35]. BAL cells were pelleted by centrifugation and the resulting supernatant (BALF) was plated onto Ashdown agar in appropriate dilutions. The BALF was stored at -70°C until use.

### Measurement of cytokines and myeloperoxidase

Quantification of cytokines (interleukin-6 (IL-6), interleukin-12 (IL-12), monocyte chemoattractant protein-1 (MCP-1), interferon γ (IFNγ) and tumor necrosis factor alpha (TNFα)) in serum and BALF was done using Cytometric Bead Array (CBA) multiplex assay (BD Biosciences, Heidelberg, Germany) and the MACSQuant Analyzer (Miltenyi Biotec, Bergisch Gladbach, Germany) according to the manufacturer’s protocols. Serum myeloperoxidase levels were determined with a commercially available ELISA kit (Hycultec, Beutelsbach, Germany).

### Data presentation and statistical analysis

Comparisons between groups were tested by Student’s *t*-test or one-way ANOVA parametric test. The Dunnett post-hoc test was used to compare each of a number of infected groups with a single non-infected control group. Comparisons between treated groups were performed with the Bonferroni post-hoc test. For survival studies Kaplan-Meier analyses followed by Log-rank (Mantel-Cox) test were conducted. Statistical analyses were done using GraphPad Prism 5.0. *P* values of < 0.05 were considered statistically significant.

## Results

### *B*. *pseudomallei* modulates the expression of major iron homeostasis-related genes and induces iron accumulation in macrophages

We have recently documented that expression of HO-1, which is essential for mobilizing cellular heme iron sources, is upregulated by *B*. *pseudomallei* infection and promotes intramacrophage bacterial growth [32].

**Fig 1:**
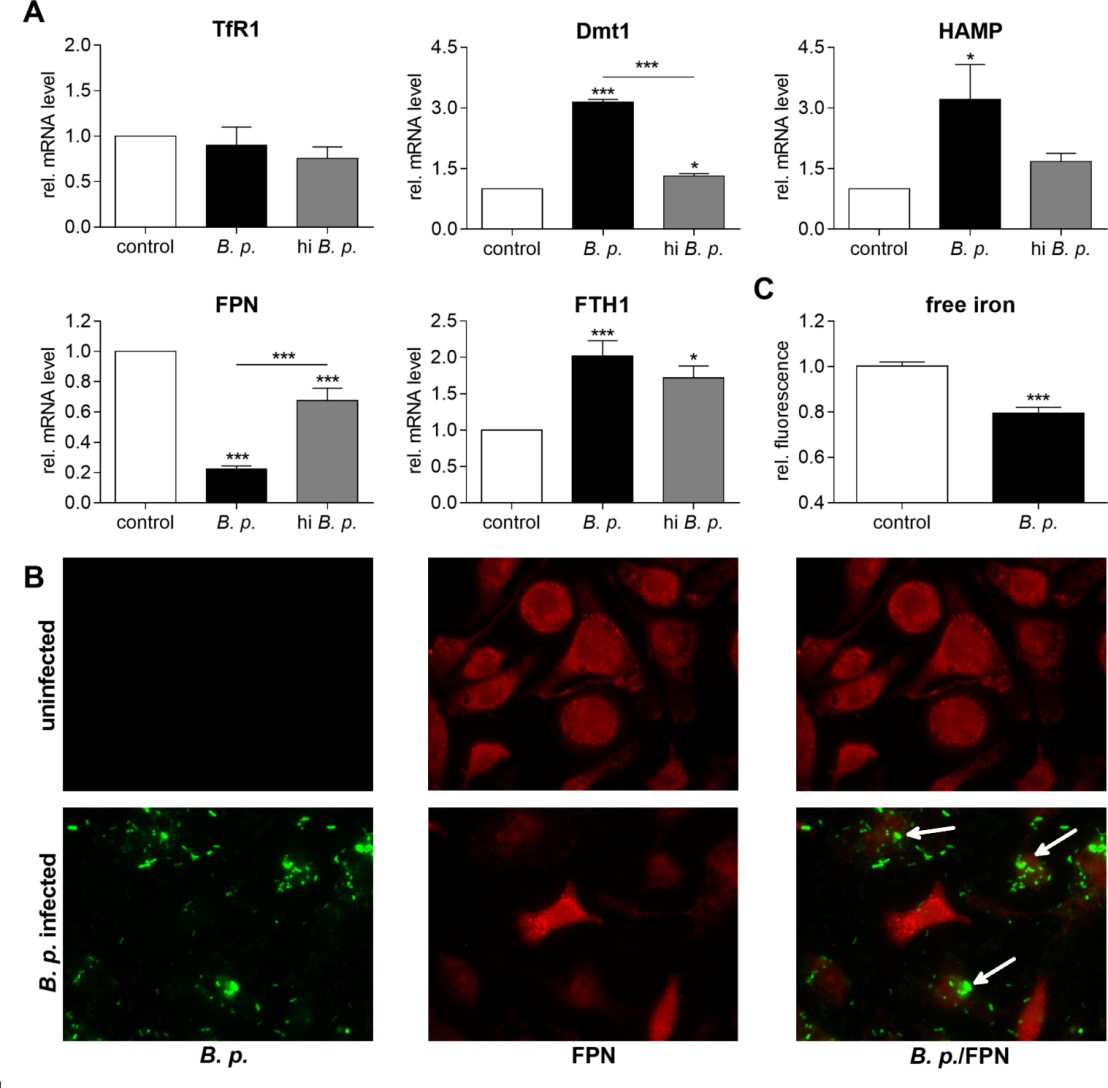
*B*. *pseudomallei* infection causes iron accumulation and ferritin upregulation in macrophages. **(A)** BMM were infected with live or heat-inactivated (hi) *B*. *pseudomallei* (*B*. *p*.) at MOI 50 and harvested 24 hours after infection. RNA was analysed for gene expression of transferrin receptor (TfR1, n = 4), divalent metal transporter-1 (Dmt1, n = 3), hepcidin antimicrobial peptide (HAMP, n = 5), ferroportin (FPN, n = 7), and ferritin heavy chain 1 (FTH1, n = 6) by qRT-PCR. Data are expressed as mean with standard error of the mean (SEM). Comparison of groups was done using one-way ANOVA and Bonferroni post-hoc test (*p<0.05, ***p<0.001). **(B)** Expression of ferroportin (red) was examined by immunocytochemical staining after 24 hours in uninfected and *B*. *pseudomallei* infected (green) BMM (magnification x1000). In the overlay (*B*. *p*./FPN) white arrows indicate decreased expression of FPN solely in cells that have ingested bacteria. **(C)** Intracellular free iron levels were monitored after 24 hours in BMM following infection with *B*. *pseudomallei* using the cell-permeable fluorescent dye Phen Green SK, which is quenched upon binding iron and thus inversely related to the labile intracellular iron accumulation. Data are presented as mean with SEM of triplicate determinations (n = 6). Statistical analyses were conducted using Student’s *t-*test (***p<0.001).

In this study, we subsequently investigated putative alterations in iron acquisition genes of macrophages in response to live and heat-inactivated *B*. *pseudomallei*, respectively. As shown in Fig 1A, no difference in mRNA levels was observed for the transferrin receptor 1 (TfR1) in the infected sample compared to the uninfected control. However, infection resulted in a 3-fold increase of divalent metal transporter-1 (Dmt1) gene expression (Figs 1A and S1). The treatment with heat-killed *B*. *pseudomallei*, added at the same number as viable bacteria, did not essentially alter mRNA expression compared to control. In order to investigate, whether infection may change macrophage iron release, we determined expression of the sole iron export protein ferroportin (Fpn). We found significantly reduced mRNA levels of Fpn in *B*. *pseudomallei*-infected compared to uninfected macrophages (Figs 1A and S1). The detected changes in Fpn mRNA levels resulted in corresponding alterations of the Fpn protein, however only in those cells that have taken up bacteria (arrows) as estimated by immunofluorescence microscopy (Fig 1B). The *B*. *pseudomallei*-driven inhibition of Fpn surface expression and iron export was expected to increase the intracellular iron content. To test this hypothesis, we measured the labile iron pool in macrophages infected with *B*. *pseudomallei* using the fluorescent probe Phen Green. Upon binding free iron, the Phen Green fluorescence is quenched and therefore inversely correlated with free intracellular iron accumulation. As shown in Fig 1C we found that the fluorescence was significantly quenched in infected compared to uninfected macrophages at 24 hours, indicating enlarged intracellular iron content. At the same time-point, infected macrophages showed a significant upregulation of the iron storage protein ferritin (ferritin heavy chain 1, Fth1) at the mRNA level, an effect also observed with heat-killed *B*. *pseudomallei* (Figs 1A and S1). Following bacterial infection transcripts of hepcidin antimicrobial peptide (Hamp) were significantly induced (Figs 1A and S1). Taken together, our data suggest that the iron accumulation in macrophages is likely to be the result of the *B*. *pseudomallei*-induced increase of Dmt1 and downregulation of Fpn expression, leading to reduced iron efflux.

### Hepcidin-mediated inhibition of the iron export function promotes iron retention and *B*. *pseudomallei* proliferation

**Fig 2:**
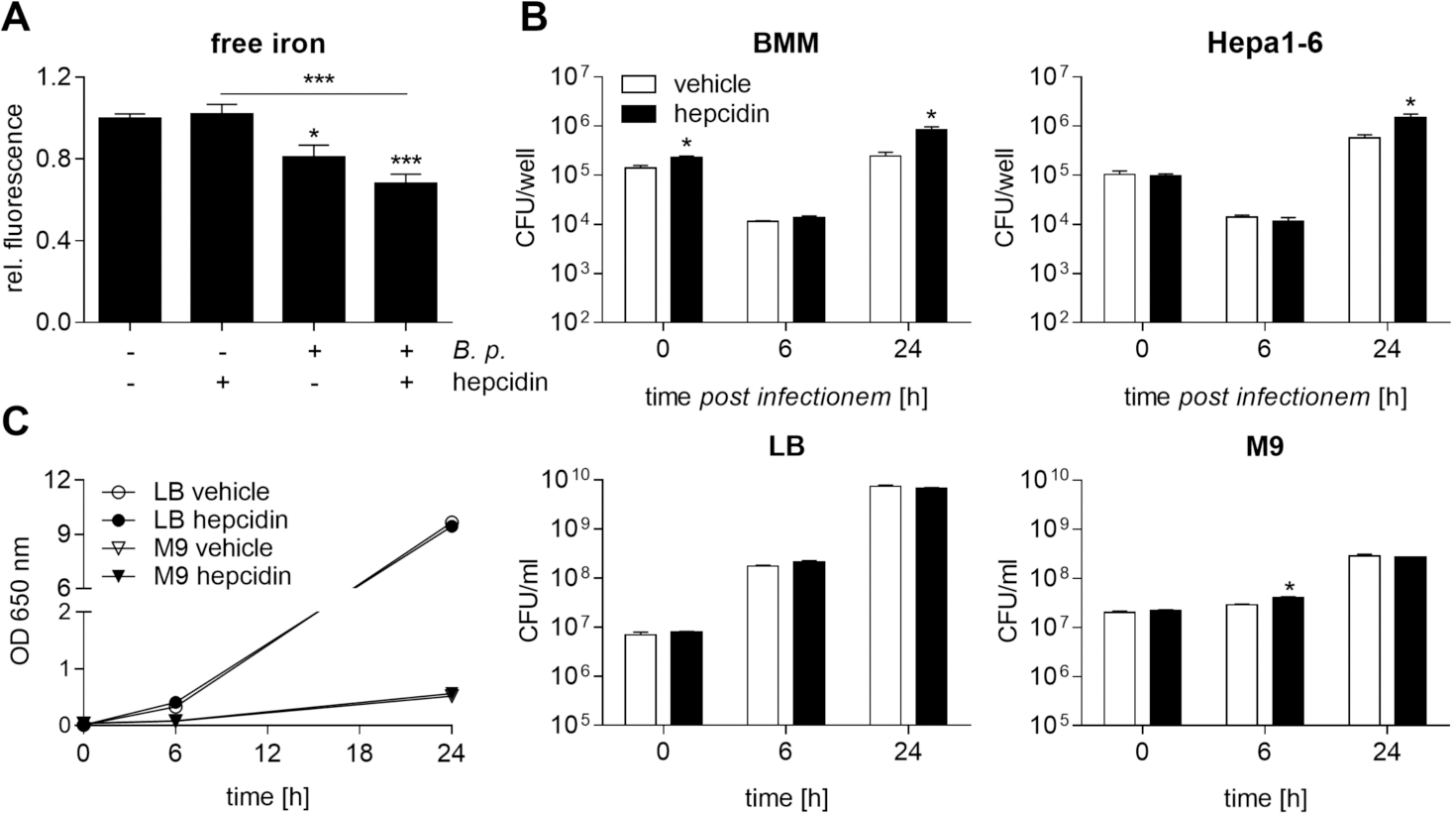
Exogenously added hepcidin leads to increased iron levels stimulating intramacrophage replication of *B*. *pseudomallei*. BMM or murine hepatoma Hepa1-6 cells were treated with hepcidin (1 μg/ml) or corresponding vehicle (A. bidest) for 20 hours followed by infection with *B*. *pseudomallei* at MOI 50 (BMM) or MOI 200 (Hepa1-6). **(A)** Intracellular free iron levels were determined after 24 hours in BMM using the iron-sensitive fluorescent probe Phen Green SK. Data are presented as mean with SEM of triplicate determinations (n = 3). Statistical analyses were performed using one-way ANOVA and the Bonferroni post-hoc test (*p<0.05, ***<0.001). **(B)** Invasion (0 h) and intracellular growth (6 h, 24 h) of *B*. *pseudomallei* were examined by kanamycin protection assay. Data are expressed as mean with SEM of triplicate determinations (BMM, n = 3; Hepa1-6, n = 4). Statistical analyses were done using Student’s *t-*test (*p<0.05). **(C)** LB broth or M9 minimal medium with or without hepcidin (1 μg/ml) or corresponding vehicle was inoculated with *B*. *pseudomallei*. The optical density (OD) at 650 nm and colony forming units (CFU)/ml were determined at indicated time points. Data are presented as mean with SEM of duplicates. Statistical analyses were performed using Student’s *t-*test (*p<0.05).

Hepcidin represents the fundamental peptide hormone that controls iron homeostasis by binding to Fpn, which causes its internalization and degradation, thus inhibiting efflux of iron [6]. We proceeded to study the impact of the Fpn-driven iron export on intracellular replication of *B*. *pseudomallei* by treating cells with hepcidin (1 μg/ml). Fig 2A shows that the relative iron quenching fluorescence was significantly decreased in *B*. *pseudomallei*-infected compared to uninfected macrophages, indicating an enlarged intracellular iron pool in response to infection at 24 hours. Higher iron levels were associated with enhanced intracellular numbers of *B*. *pseudomallei* 24 hours after infection (Fig 2B). Since hepatocytes have a large capacity for iron storage and are major producers of hepcidin, we also analysed the consequences of hepcidin administration in murine hepatoma Hepa1-6 cells. In line with macrophages, hepcidin led to higher bacterial load in hepatoma cells (Fig 2B). To exclude direct effects of hepcidin or vehicle on *B*. *pseudomallei* we performed growth kinetics in LB broth and M9 minimal medium. However, bacterial growth was not affected by hepcidin in both media at 24 hours (Fig 2C). Thus, our results indicate that the hepcidin-mediated intracellular iron retention is beneficial for the establishment of *B*. *pseudomallei* infection in host macrophages.

### Administration of iron attenuates immune defense mechanisms of macrophages improving survival of *B*. *pseudomallei*

**Fig 3:**
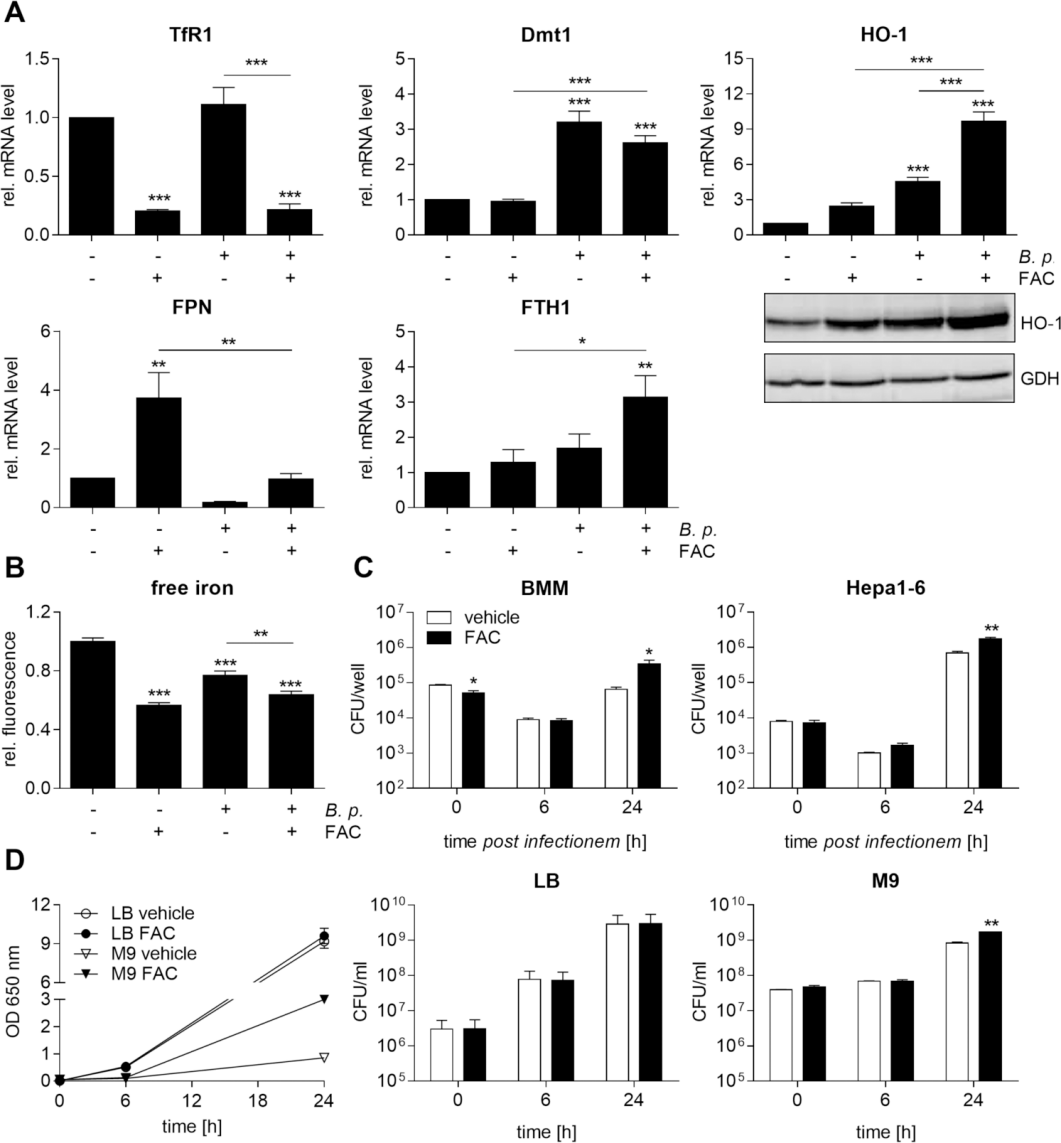
Iron loading promotes HO-1 and FTH1 expression, intracellular iron availability and *B*. *pseudomallei* growth. BMM were treated with ferric ammonium citrate (FAC, 100 μM) or corresponding vehicle (A. bidest) for 20 hours followed by infection with *B*. *pseudomallei* at MOI 50. **(A)** 24 hours after infection gene expression of TfR1 (n = 6), Dmt1 (n = 5), HO-1 (n = 6), FPN (n = 5), and FTH1 (n = 6) was analysed by qRT-PCR. Expression of HO-1 was detected by immunoblot in cell lysates. Data are presented as mean with SEM. **(B)** Intracellular free iron levels were determined after 24 hours using the iron-sensitive fluorescent probe Phen Green SK. Data are expressed as mean with SEM of triplicate determinations (n = 3). (A, B) Comparison of groups was done using one-way ANOVA and the Bonferroni post-hoc test (*p<0.05, **p<0.01, ***p<0.001). **(C)** BMM or murine hepatoma Hepa1-6 cells were treated with FAC (100 μM) or corresponding vehicle for 20 hours followed by infection with *B*. *pseudomallei* at MOI 50 (BMM) or MOI 200 (Hepa1-6). Invasion (0 h) and intracellular growth (6 h, 24 h) of *B*. *pseudomallei* were examined by kanamycin protection assay. Data are presented as mean with SEM of triplicate determinations (n = 3). **(D)** LB broth or M9 minimal medium with or without FAC (100 μM) or corresponding vehicle was inoculated with *B*. *pseudomallei*. The optical density (OD) at 650 nm and colony forming units (CFU)/ml were determined at indicated time points. Data are presented as mean and SEM of duplicates. (C, D) Statistical analyses were conducted using Student’s *t-*test (*p<0.05, **p<0.01).

Next we analysed the consequences of iron supplementation on transcription of iron homeostasis-related genes as well as iron content in macrophages infected with *B*. *pseudomallei*. The delivery of non-transferrin bound iron, provided as ferric ammonium citrate (FAC, 100 μM), strongly reduced expression of TfR1 in both infected and uninfected macrophages as expected, whereas expression of Dmt1 was not significantly changed by addition of FAC (Fig 3A). In agreement with previous studies, showing Fpn upregulation in response to iron supplementation [37], FAC (Fig 3A) or ferrous sulphate (FeSO_4_; S2A Fig) increased Fpn gene expression in uninfected cells. However, upon infection with *B*. *pseudomallei*, Fpn mRNA levels were significantly reduced in both iron- and solvent-treated macrophages, whereas Fth1 and HO-1 expression were significantly enhanced by FAC (Figs 3A and S2A). Loading of macrophages with the iron sources resulted in a significant reduction of the iron quenching fluorescence suggesting a higher intracellular iron accumulation 24 hours after infection (Figs 3B and S2B). In addition, we determined the invasion and the course of intracellular bacterial burden in FAC/FeSO_4_- and vehicle-treated macrophages as well as in hepatoma Hepa1-6 cells at different time points after *B*. *pseudomallei* infection. As shown in Figs 3C and S2C, the FAC- or FeSO_4_-driven iron accumulation in cells 24 hours after infection was associated with an increase in intracellular bacterial numbers. Whereas FAC or FeSO_4_ did not change the growth rate of *B*. *pseudomallei* in LB broth, the bacterial growth was significantly improved in iron-free M9 minimal medium supplemented with FAC or FeSO_4_ (Figs 3D and S2D). Our data indicate that FAC or FeSO_4_ treatment, resulting in a rise of the intracellular iron pool, facilitates intracellular growth of *B*. *pseudomallei*.

**Fig 4:**
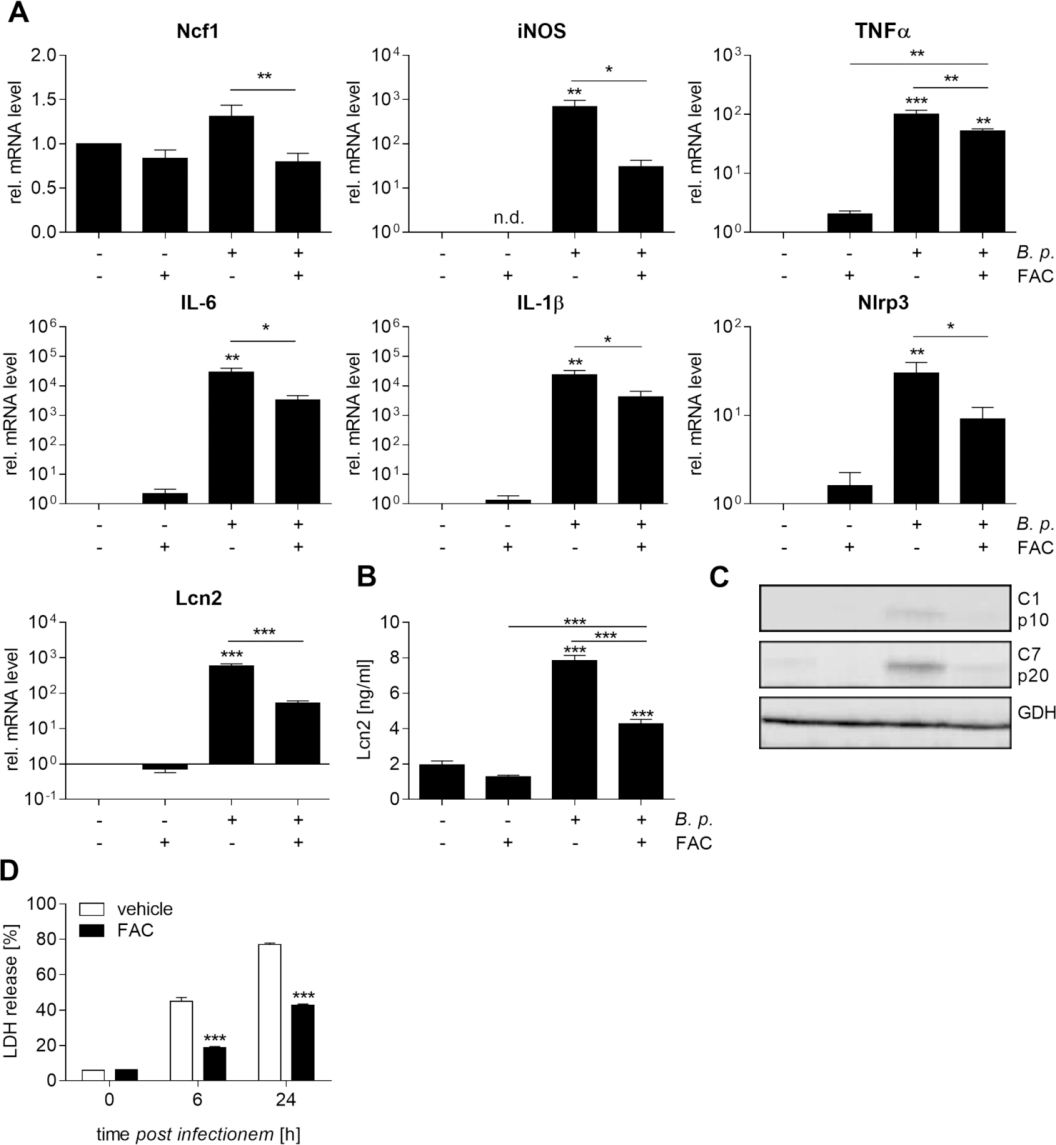
Iron supplementation impairs immune response pathways in *B*. *pseudomallei*-infected macrophages. BMM were treated with FAC (100 μM) or corresponding vehicle (A. bidest) for 20 hours followed by infection with *B*. *pseudomallei* at MOI 50. **(A)** 24 hours after infection gene expression of neutrophil cytosolic factor 1 (Ncf1, n = 6), inducible nitric oxide synthase (iNOS, n = 4), tumor necrosis factor alpha (TNFα, n = 5), interleukin-6 (IL-6, n = 5), interleukin-1β (IL-1β, n = 4), NLR family pyrin domain containing 3 (Nlrp3, n = 4), and lipocalin 2 (Lcn2, n = 6) was analysed by qRT-PCR. **(B)** Lcn2 secretion in supernatants was measured by ELISA (n = 3). (A, B) Data are expressed as mean with SEM. Comparison of groups was done using one-way ANOVA and the Bonferroni post-hoc test (*p<0.05, **p<0.01, ***p<0.001). **(C)** Cleavage of caspase-1 and -7 and expression of GAPDH were detected by immunoblot in cell lysates of FAC- or vehicle-treated BMM at 24 hours after infection with *B*. *pseudomallei*. **(D)** Cytotoxicity was measured as lactate dehydrogenase (LDH) release in cell supernatants of FAC- or vehicle-treated *B*. *pseudomallei*-infected BMM. Data are presented as mean with SEM of triplicate determinations (n = 3). Statistical analyses were conducted using Student’s *t-*test (***p<0.001).

Based on these results, we then asked whether iron supplementation may impact the efficiency of macrophage immune effector functions in the course of *Burkholderia* infection, such as the generation of reactive oxygen or nitrogen species, inflammatory mediators, antimicrobial protein expression and cell death induction as well. Our data show that expression of neutrophil cytosolic factor 1 (Ncf1, p47phox, NOXO2), a component of the NADPH oxidase complex, as well as inducible nitric oxide synthase (iNOS) is strongly reduced in response to FAC in infected macrophages (Fig 4A). Furthermore, we found that iron loading was paralleled by diminished mRNA levels of inflammatory cytokines including tumor necrosis factor alpha (TNFα), interleukin-6 (IL-6) and interleukin-1β (IL-1β) as compared to infected control cells. Importantly, upon *B*. *pseudomallei* infection, FAC-treated macrophages showed a significantly lower expression and secretion of the antimicrobial siderophore scavenging peptide lipocalin 2 (Lcn2) (Fig 4A and 4B). Since activation of the caspase-1 inflammasome is an important host defense mechanism against *B*. *pseudomallei* [35], we evaluated the expression of NLR family pyrin domain containing 3 (Nlrp3), processing of caspase-1 as well as release of lactate dehydrogenase (LDH) in cell supernatants. Treatment of macrophages with FAC prior to infection exerted inhibitory effects towards the expression of Nlrp3 (Fig 4A) and resulted in less cleavage of both caspase-1 and downstream caspase-7 (Fig 4C) compared to the solvent. Minor activation of caspase-1 in response to iron was accompanied by a reduced induction of pyroptosis, a highly proinflammatory form of cell death (Fig 4D). Thus, our data provide evidence that iron supplementation downregulates antibacterial immune effector pathways of macrophages promoting *B*. *pseudomallei* proliferation.

### Limitation of iron availability improves control of *B*. *pseudomallei* growth in macrophages

**Fig 5:**
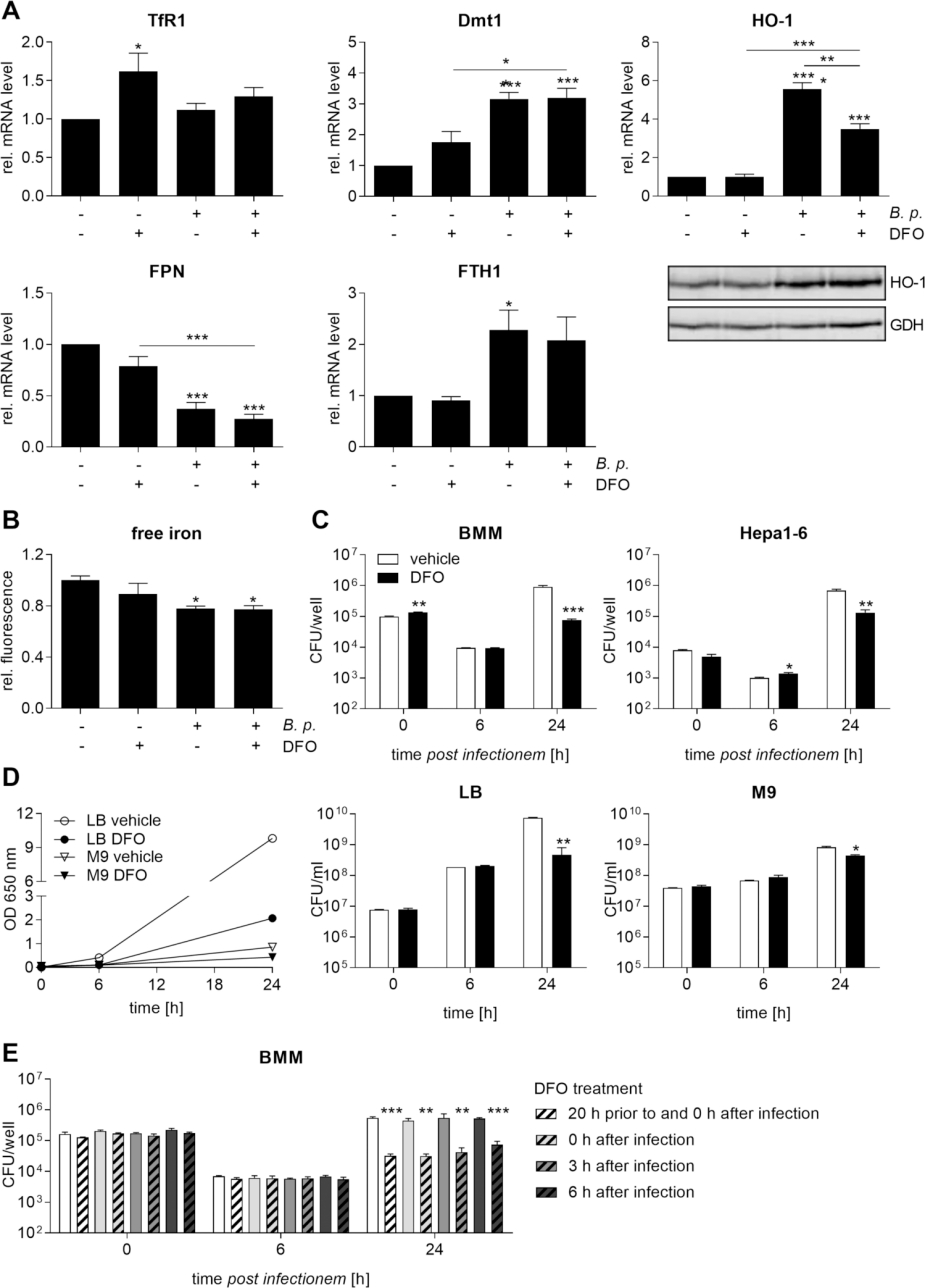
Iron chelation limits intracellular *B*. *pseudomallei* growth by direct bacteriostatic properties. BMM were treated with deferoxamine (DFO, 50 μM) or corresponding vehicle (A. bidest) for 20 hours followed by infection with *B*. *pseudomallei* at MOI 50. **(A)** 24 hours after infection gene expression of TfR1 (n = 8), Dmt1 (n = 8), HO-1 (n = 6), FPN (n = 6), and FTH1 (n = 5) was analysed by qRT-PCR. Expression of HO-1 was detected by immunoblot in cell lysates. Data are expressed as mean with SEM. **(B)** Intracellular free iron levels were determined after 24 hours using the iron-sensitive fluorescent probe Phen Green SK. Data are presented as mean with SEM of triplicate determinations (n = 3). (A, B) Comparison of groups was done using one-way ANOVA and the Bonferroni post-hoc test (*p<0.05, **p<0.01, ***p<0.001). **(C)** BMM or murine hepatoma Hepa1-6 cells were treated with DFO (50 μM) or corresponding vehicle for 20 hours followed by infection with *B*. *pseudomallei* at MOI 50 (BMM) or MOI 200 (Hepa1-6). Invasion (0 h) and intracellular growth (6 h, 24 h) of *B*. *pseudomallei* were examined by kanamycin protection assay. Data are shown as mean with SEM of triplicate determinations (BMM, n = 3; Hepa1-6, n = 4). Statistical analyses were done using Student’s *t-*test (*p<0.05, **p<0.01, ***p<0.001). **(D)** LB broth or M9 minimal medium with or without DFO (50 μM) or corresponding vehicle was inoculated with *B*. *pseudomallei*. The optical density (OD) at 650 nm and colony forming units (CFU)/ml were determined at indicated time points. Data are expressed as mean and SEM of duplicates. Statistical analyses were conducted using Student’s *t-*test (*p<0.05, **p<0.01). **(E)** BMM were exposed to DFO (50 μM, dashed bars) or vehicle (unfilled bars) as indicated and infected with *B*. *pseudomallei*. Treatment was carried out both 20 hours prior to and directly (0 h) after infection, directly (0 h), three hours (3 h), or six hours (6 h) after infection. Intracellular bacterial growth was examined by kanamycin protection assay at 0, 6, and 24 hours. Data are presented as mean with SEM of triplicate determinations (n = 2). Statistical analyses were done using Student’s *t-*test (**p<0.01, ***p<0.001).

In further studies we determined the transcriptional responses of macrophages to the iron-chelating agent deferoxamine (DFO, 50 μM). As shown in Fig 5A, DFO increased the expression of TfR1 (*p<0.05) and showed a trend towards higher Dmt1 mRNA levels in uninfected macrophages, whereas expression of both genes was not affected in infected cells by the DFO treatment. Iron chelation led to slightly decreased Fpn mRNA levels in both uninfected and infected cells at 24 hours and significantly diminished *B*. *pseudomallei*-mediated HO-1 gene induction as well. The HO-1 protein expression in infected macrophages remained unchanged in response to DFO. Furthermore, no difference in Fth1 mRNA or intracellular iron levels (Fig 5B) could be detected after treatment with DFO. In order to examine whether *B*. *pseudomallei* can survive intracellularly after DFO administration, we determined the viability of *B*. *pseudomallei* during infection of macrophages and hepatoma cells. Whereas both uptake of bacteria into cells and bacterial load at 6 hours after infection was only slightly affected in cells exposed to DFO compared to vehicle, the number of bacteria recovered after 24 hours of infection was one log lower following treatment with DFO (Fig 5C). In subsequent experiments we tested the consequences of DFO pretreatment versus posttreatment on intracellular *B*. *pseudomallei* growth. Thus, macrophages were exposed to DFO or vehicle 20 hours prior to infection and directly after infection (pretreatment) as described above. Posttreatment was performed by the addition of DFO or vehicle directly, 3, or 6 hours after infection. Fig 5E indicates that intracellular bacterial counts were significantly lower in DFO- compared to vehicle-treated macrophages irrespective of the time of treatment. Finally, we investigated the effect of the iron chelator on *B*. *pseudomallei* growth in LB broth and M9 minimal broth. We found that both the optical density and CFU/ml were strongly reduced by DFO in the nutritionally rich LB medium at 24 hours, whereas *B*. *pseudomallei* growth inhibition in M9 minimal medium was less pronounced (Fig 5D).

Analogous to our iron supplementation studies, we compared the immune defense functions in macrophages in response to the iron chelating drug. S3A Fig shows that gene expression of Ncf1, iNOS, IL-6, IL-1β, and Lcn2 was not significantly changed, whereas the TNFα transcript and Lcn2 secretion (S3B Fig) were slightly reduced in response to DFO in infected macrophages compared to the vehicle. Treatment of infected cells with DFO did neither influence mRNA expression of Nlrp3 (S3A Fig) nor cleavage of caspase-1 and caspase-7 (S3C Fig). However, although caspase-1 activation was not altered, the LDH release was surprisingly increased by DFO both 6 and 24 hours after infection (S3D Fig), which probably points to a cytotoxic effect of DFO. Taken together, our results suggest that DFO treatment attenuates intracellular growth of *B*. *pseudomallei* not via modulation of immune effector mechanisms but rather by direct bacteriostatic properties.

### Infection of mice with *B*. *pseudomallei* leads to intracellular iron withhold and hypoferremia

**Fig 6:**
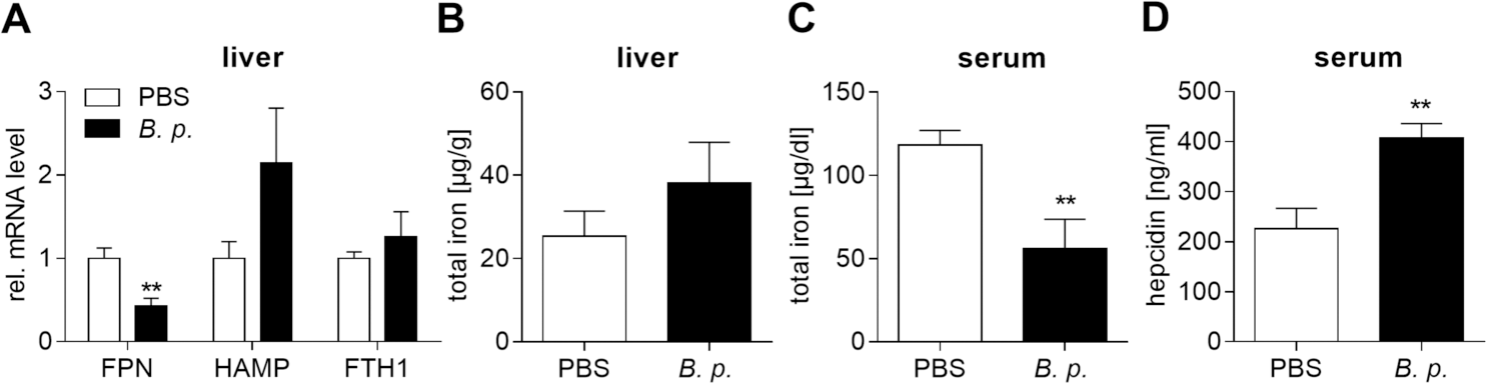
Infection of mice with *B*. *pseudomallei* leads to increased hepatic iron storage and reduced serum iron content. C57BL/6 mice were intranasally inoculated with *B*. *pseudomallei* at 500 CFU. **(A)** Gene expression of FPN, HAMP and FTH1 in the liver was analysed by qRT-PCR 24 hours after infection. Data are expressed as mean with SEM (n = 7). The relative expression of target genes was normalized to the mean of the control, which was set to one. Statistical analyses were done using Student’s *t-*test (**p<0.01). Total iron concentration **(B)** in the liver and **(C)** in serum and **(D)** release of hepcidin in serum were determined 48 hours after infection. Data are presented as mean with SEM (n = 8). Statistical analyses were conducted using Student’s *t-*test (*p<0.05, **p<0.01).

We further analysed the expression of iron-related genes in response to intranasal infection of C57BL/6 mice with *B*. *pseudomallei*. 24 hours after infection, Hamp mRNA levels were increased (not significant) and Fpn mRNA levels were significantly decreased in the liver in response to *B*. *pseudomallei*, whereas Fth1 expression was not different (Fig 6A). Moreover, we found an elevated hepatic iron concentration (not significant; Fig 6B) associated with higher systemic hepcidin (p<0.01; Fig 6D) and lower systemic iron levels (p<0.01; Fig 6C), indicating hypoferremic conditions.

In order to determine the effect of iron supplementation or depletion during *in vivo* infection, we treated mice with FAC (5 mg/kg), DFO (100 mg/kg) or vehicle by intraperitoneal injection followed by pulmonary infection with *B*. *pseudomallei*. As shown in S4A Fig, repeated administration of FAC resulted in slightly elevated gene expression of Hamp and Fth1, and reduced mRNA levels of Fpn 48 hours after infection, although results were not statistically significant. Intracellular iron concentration in the liver (S4B Fig) as well as serum hepcidin concentration (S4D Fig) were unchanged, whereas serum iron levels were significantly increased (p<0.01; S4C Fig). Conversely, deficiency of iron by DFO slightly reduced gene expression of Hamp, increased Fpn expression but did not change ferritin expression (not significant; S4A Fig). Treatment with DFO diminished total hepatic iron content (p<0.05; S4B Fig), systemic iron levels (not significant; S4C Fig) as well as hepcidin levels (p<0.01; S4D Fig). Our data show that iron levels are increased by iron supplementation and decreased by iron chelation.

### Iron deficiency improves outcome during experimental melioidosis

**Fig 7:**
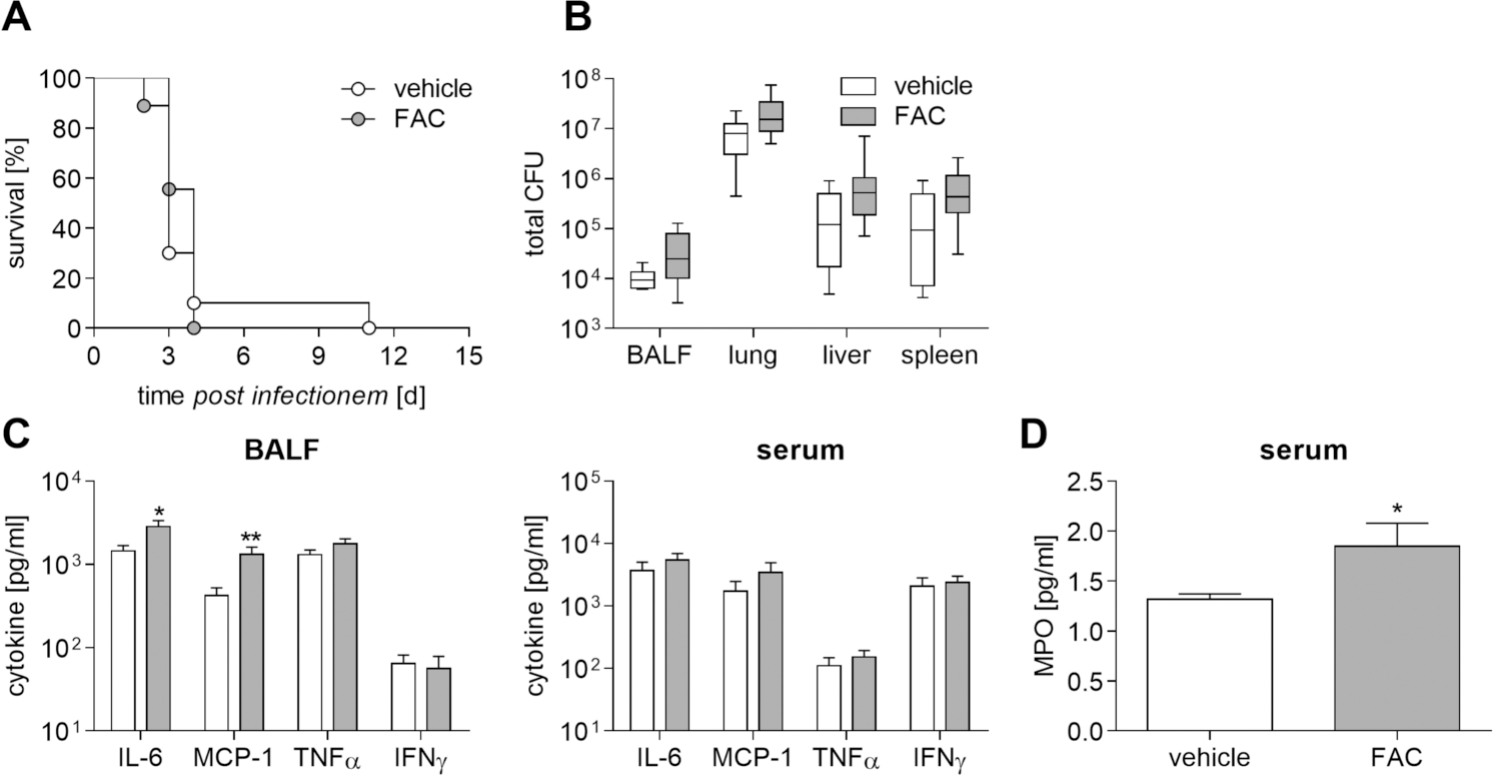
Iron availability promotes bacterial growth at the primary site of infection and increases the inflammatory response. FAC (5 mg/kg) or vehicle (D-PBS)-treated C57BL/6 mice were intranasally inoculated with *B*. *pseudomallei* at 500 CFU. **(A)** Cumulative survival rate between groups was compared using Log-rank Kaplan-Meier test (2 independent experiments, n = 10). **(B)** 48 hours after infection, the bacterial load (CFU) in BALF (n = 8) and organs (n = 10) was determined. Data from two experiments are expressed as box and whisker plots indicating minimum, maximum, quartiles and median. **(C)** Cytokine production (IL-6, MCP-1, TNFα, IFNγ) in BALF (n = 8) and serum (n = 10), and **(D)** myeloperoxidase (MPO) levels in serum (n = 5) were measured 48 hours after infection. (C, D) Data from two experiments are presented as mean with SEM. (B-D) Statistical analyses were done using a Student’s *t-*test (*p<0.05, **p<0.01).

Finally, we verified the impact of either iron supplementation or deprivation on survival of *B*. *pseudomallei* in our respiratory melioidosis model. Administration of FAC to mice resulted in only marginally enhanced bacterial numbers in bronchoalveolar lavage fluids (BALF) as well as in organs compared to vehicle-treated mice 48 hours after infection (Fig 7B) that were not leading to a higher mortality rate of iron-supplemented mice (Fig 7A). To further characterize effects of *in vivo* iron loading, we examined cytokine secretion in both BALF and serum of FAC- and vehicle-treated mice 48 hours after infection. We found solely increased levels of IL-6 and monocyte chemoattractant protein 1 (MCP-1, CCL-2) in BALF, but not in serum, whereas secretion of TNFα and IFNγ were neither changed in BALF nor serum by FAC (Fig 7C). In addition, we observed that recruitment of neutrophils in serum, as determined by myeloperoxidase (MPO) levels, was significantly higher in FAC-treated mice (Fig 7D). This might indicate that conditions with increased iron stores are likely to be a risk factor and thus increase susceptibility to melioidosis.

**Fig 8:**
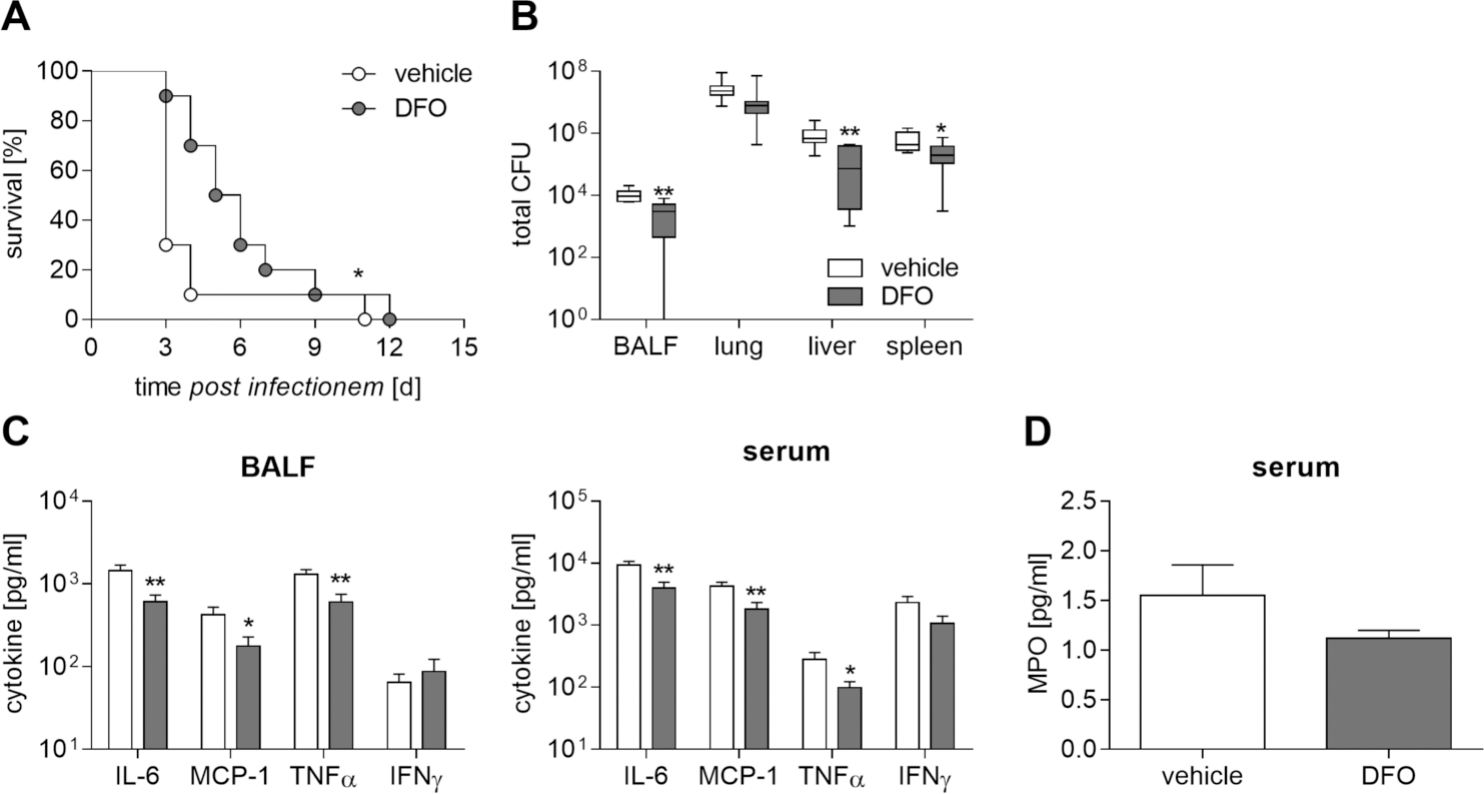
Limitation of iron availability ameliorates bacterial clearance and survival of infected mice. DFO (100 mg/kg) or vehicle (D-PBS)-treated C57BL/6 mice were intranasally inoculated with *B*. *pseudomallei* at 500 CFU. **(A)** Cumulative survival rate between groups was compared using (Log-rank Kaplan-Meier test, *p<0.005 compared to vehicle-treated mice (2 independent experiments, n = 10)). **(B)** 48 hours after infection, the bacterial load (CFU) in BALF (n = 8) and organs (n = 10) was determined. Data from two experiments are expressed as box and whisker plots indicating minimum, maximum, quartiles and median. **(C)** Cytokine production (IL-6, MCP-1, TNFα, IFNγ) in BALF (n = 8) and serum (n = 10), and **(D)** myeloperoxidase (MPO) levels in serum (n = 5) were measured 48 hours after infection. (C, D) Data from two experiments are presented as mean with SEM. (B-D) Statistical analyses were done using a Student’s *t-*test (*p<0.05, **p<0.01).

Subsequent studies indicated that *in vivo* administration of the iron chelator DFO causing lower hepatic and systemic iron levels (S4B and S4C Fig) diminishes bacterial load in BALF, lung, liver, and spleen (Fig 8B) and was associated with significantly improved survival of mice (Fig 8A). Both BALF and serum levels of IL-6, MCP-1 and TNFα, but not of IFNγ were attenuated (Fig 8C). The MPO concentration in serum was slightly reduced in iron-depleted mice as well (Fig 8D). In conclusion, our *in vivo* data suggest that an iron chelation therapy might influence clinical disease.

## Discussion

The major strategy of macrophages to limit iron availability to intracellular pathogens involves stimulation of cellular iron export together with attenuation of iron import. However, the present study demonstrates that the facultative intracellular bacterium *B*. *pseudomallei* is able to evade these immune defense strategies by modulation of iron homeostasis in murine macrophages. Own previous work revealed that infection of host cells with *Burkholderia* induces expression of HO-1, which is involved in degradation of heme producing CO, biliverdin, and ferrous iron, and appears to be beneficial for intracellular bacterial survival [32]. Among various mechanisms, this might also be an attempt of the pathogen to ensure a sufficient availability of iron.

In this study we now found an upregulation of Dmt1, a divalent metal transporter, which is located in plasma and/or phagosomal membranes, promoting the internalization of non-transferrin bound iron into cells and/or its transport from the phagosome into the cytosol. Although, *B*. *pseudomallei* can acquire the metal from transferrin via malleobactin [16–18], expression of TfR1 was unchanged following infection. Similar observations have been made with *Listeria monocytogenes* exhibiting a closely related intracellular lifestyle [38]. The cytosolic pathogen *Francisella tularensis* initiates an iron acquisition program by induction of both Dmt1 and TfR1 with a substantially increase in the host cell labile iron pool [39], and several parasites such as *Leishmania donovani* and *Toxoplasma gondii* enhance expression of TfR1 during infection as well [40, 41]. However, challenge of macrophage-like cells with *Salmonella*, which resides in phagosomes, does not require Dmt1 or TfR1 expression for effective intracellular survival [39, 42], although an induction of Dmt1 expression has been described in primary macrophages [43]. These data indicate distinct regulatory patterns of macrophage iron import during infection, which might be reliant on the cell type, the pathogen and its intracellular localization.

Apart from improved iron uptake, we further provide evidence that *B*. *pseudomallei* interferes with cellular iron release by downregulating the exclusive exporter Fpn and upregulating transcription of Hamp leading to an increase of intramacrophage labile iron pool. In accordance, some pathogens have been shown to induce their macrophages to secrete hepcidin and to degrade Fpn in a TLR4-dependent manner stimulating cellular iron retention [37, 44, 45]. Hepcidin, although mainly produced in the liver [46], can also be produced locally by macrophages in an autocrine manner after infection [44]. We found that the *B*. *pseudomallei*-mediated downregulation of Fpn occurs within 18 hours, while the induced transcription of hepcidin becomes apparent only at 24 hours. This suggests that regulation of Fpn expression is at least in part hepcidin-independent and might be regulated by other factors. A recent study revealed that TLR2/TLR6 activation can trigger a strong decline in Fpn expression in murine liver, spleen, and macrophages, without altering hepcidin levels indicating that rapid hepcidin-independent Fpn downregulation may be a first-line response to limit iron access for pathogens [47]. Contrary to *B*. *pseudomallei*, both *L*. *monocytogenes* and *S*. *enterica* serovar Typhimurium [38, 42] trigger Fpn gene induction in macrophage-like cells, and *Chlamydia psittaci* or *Legionella pneumophila* do not change Fpn levels in primary macrophages [48]. Following infection with *Mycobacterium* Fpn mRNA expression appears to be negatively or positively regulated in several mouse macrophage populations, which may refere to the different basal levels of Fpn mRNA in unstimulated cells [49].

In accordance with previous evidence arising from studies with several pathogens [37, 42, 43, 50], *B*. *pseudomallei* favors the expression of the cytosolic iron storage protein ferritin. This induction was not dependent on the intracellular presence of viable *B*. *pseudomallei* as heat-inactivated bacteria were sufficient to trigger ferritin upregulation observed in response to living microbes and might be consistent with the role for LPS in ferritin stimulation. Kvitko et al. [23] proposed that *B*. *pseudomallei* is capable of acquiring the metal from ferritin as an alternate source most likely by a secreted protease as previously shown for *B*. *cenocepacia* [51]. Both *N*. *meningitidis* [52] and *S*. *enterica* serovar Typhimurium can utilize ferritin-derived iron for their intracellular proliferation, although *Salmonella*-infected macrophages seem to reduce the integration of iron into ferritin to withhold iron from the pathogen [42].

In the present work we observed decreased expression of Fpn in the liver after systemic spread of *B*. *pseudomallei* in a pulmonary melioidosis model associated with elevated hepcidin concentrations in serum as well as higher hepatic and lower systemic iron levels indicating hypoferremic conditions. These results are in agreement with our *in vitro* data from primary macrophages and different bacterial models [44, 53–56]. Thus, we propose that the *B*. *pseudomallei*-driven changes in expression of host genes implicated in iron import, storage and export might be a strategy of the pathogen to facilitate iron access and to promote its intracellular survival.

The requirement of iron for intracellular *B*. *pseudomallei* growth has initially been documented in iron-laden lung epithelial A549 cells [57]. We now demonstrate that treatment of host cells with hepcidin or iron supplements increases cytosolic iron availability and improves *B*. *pseudomallei* replication in both macrophages and hepatoma cells. Due to an excess presence of labile iron, triggered by increased HO-1 and Dmt1 expression, ferritin degradation or still unknown mechanisms, hepcidin does impact iron availability mainly in *B*. *pseudomallei*-infected macrophages. In line with previous observations, showing upregulation of Fpn, Fth1 and HO-1 in response to iron loading of macrophages [37, 58], infection with *Burkholderia* downregulates Fpn transcription in both iron- and vehicle-treated macrophages, whereas Fth1 and HO-1 mRNA levels are significantly upregulated by FAC or FeSO_4_. Treatment with iron supplements improves bacterial growth in minimal medium, but hepcidin does not affect the growth rate at 24 hours when added directly to bacterial cultures, indicating that an interaction with host cells is necessary. Stimulation of Fpn functionality, limiting the availability of the metal, restricted the intracellular growth of bacterial pathogens, whereas Fpn degradation upon hepcidin treatment enhanced iron supply in the cytosol and was associated with pathogen replication [37, 38, 48, 59]. A recent study described that prolonged iron overload results in alkalization of lysosomes, affects the organelle function and autophagic flux of human macrophage-like cells, and leads to lower bactericidal efficiency against *P*. *aeruginosa*. Cotreatment with the iron chelator DFO was able to restore the lysosomal acidification and antibacterial activity of host cells [60]. Thus, inhibition of hepcidin expression or depletion of macrophage iron levels might be therapeutic since they could decrease intracellular bacterial growth. Reports with cultured cells have revealed that addition of DFO leads to reduced proliferation of a number of intracellular bacteria [42, 48]. Accordingly, treatment with the iron chelator markedly impaired *B*. *pseudomallei* replication not only in nutritionally rich and minimal medium, but also in macrophages and hepatoma cells. However, the mechanisms accounting for its beneficial effects on *Burkholderia* infection are not yet clear and appear to be independent of host iron metabolism or immune defense pathways. Thus, we suggest that DFO exerts direct bacteriostatic action on the pathogen.

Macrophages deficient for HO-1 function have been shown to restrict intracellular iron levels during infection with *Salmonella* and to enhance production of reactive oxygen species which promote NFκB-mediated activation of a proinflammatory immune response (e.g. TNFα, iNOS, p47phox) leading to reduced survival of *Salmonella* [43]. Conversely, cellular iron retention was associated with reduced cytokine expression by macrophages and impaired pathogen control [61, 62]. We recently found that *B*. *pseudomallei*-driven upregulation of HO-1 in macrophages is probably not only a strategy to ensure efficient intracellular iron supply, but also an attempt to weaken host-derived antibacterial effector mechanisms such as the inflammatory response for establishment of infection [32]. This assumption could be strengthened by the present findings, that increased iron supply in FAC-treated macrophages markedly reduces antimicrobial effector pathways of macrophages including expression of free radical generating enzymes and inflammatory cytokines (e.g. IL-1β), caspase-1-mediated pyroptotic cell death, or production of the antimicrobial factor Lcn2. The NADPH oxidase-derived production of reactive oxygen species has been shown to contribute to suppression of *B*. *pseudomallei* growth in murine macrophages and human monocytes [63, 64]. Whereas, iNOS appears to be dispensable for host resistance of C57BL/6 macrophages against the pathogen [63–65], nitric oxide is able to kill aerobic, anaerobic and persistent *B*. *pseudomallei* [66]. The pathogen also induces a rapid caspase-1-dependent macrophage death that effectively restricts the intracellular bacterial survival [35, 67, 68]. Lipocalin-2 represents an essential component of the antimicrobial innate immune system. It binds and neutralizes bacterial siderophores such as enterobactin produced by *S*. *enterica* serovar Typhimurium, *E*. *coli* or *Klebsiella pneumoniae* as well as carboxymycobactins released by mycobacteria [69–72]. Lcn2 was reported to affect host iron metabolism by suppressing the intracellular formation of ferritin, to chemoattract neutrophils and to have immunomodulatory effects as well. Lcn2 seems to be involved in host defense against *Chlamydia pneumoniae* and *S*. *enterica* serovar Typhimurium by limiting the availability of iron to the pathogen [73, 74]. In contrast, in a pneumococcal infection model, Lcn2 expression by neutrophils resulted in decreased survival of mice [75]. However, its role during melioidosis remains elusive and needs further investigation.

Melioidosis is connected with basic medical disorders such as diabetes mellitus and renal disease. Several lines of evidence propose that conditions with iron overload such as thalassemia are related to disease as well [24, 27–31]. A recent study indicated a higher incidence of melioidosis in Malaysia among children with thalassemia that significantly decreased with intravenous iron chelation therapy [25]. Consistent with these observations during human melioidosis, we established in a murine experimental model that FAC-derived iron supplementation tends to stimulate both systemic and hepatic iron load leading to higher bacterial numbers in organs as well as release of cytokines and MPO. On the other hand, the DFO-mediated limitation of iron availability improves outcome of *B*. *pseudomallei*-infected mice as shown by reduced bacterial burden and inflammatory response.

Thus, it is reasonable to propose that manipulation of host iron metabolism or direct targeting of iron may represent a new therapeutic approach during melioidosis. In this context, Nifedipine, a calcium channel blocker, can induce Fpn expression, thus mobilizing tissue iron and improving host resistance to *Salmonella* [76]. Recent evidence further suggest that an inverse agonist of estrogen related receptor gamma (ERRγ) is able to ameliorate *Salmonella*-induced hypoferremia by reduction of ERRγ-mediated hepcidin expression in hepatocytes leading to a better control of infection [55]. This is in line with findings demonstrating that control of the hepcidin-Fpn-axis effects on intracellular *Salmonella* proliferation and the outcome in mice [62]. Since HO-1 may affect iron availability for intracellular pathogens, pharmacological targeting of heme catabolism may also be of relevance to modulate host iron homeostasis and iron trafficking to combat infection. We previously found that HO-1 gene knockdown in macrophages results in impaired survival of *B*. *pseudomallei* [32]. Consistent, depletion of HO-1 has been shown to cause an induction of Fpn-mediated iron efflux in macrophages, limiting availability of iron for *Salmonella*, and to stimulate antimicrobial immune effector pathways including radical formation, that can be attributed to improved control of infection [43]. Moreover, iron chelators might also be useful for the treatment of infections with intracellular pathogens. In addition to our findings in primary macrophages and mice using deferoxamine, the oral iron chelator deferasirox is thought to have beneficial effects on *Salmonella* infection *in vitro* and *in vivo* [61]. Both deferasirox and deferriprone have been shown to remove the metal from iron-loaded macrophages limiting intracellular *C*. *psittaci* and *L*. *pneumophila* growth [48]. However, whether these results obtained with oral iron chelators can be translated into clinical benefits for infected humans remains to be examined.

In summary, the results of the present study contribute to a much better understanding of adaptive changes of the host iron metabolism following *B*. *pseudomallei* infection and provide novel insights into the important role played by cytosolic iron pools and/or ferritin for establishment of intracellular infection. This opens the way for future investigations of how the pathogen might use iron sequestered in ferritin, and whether modulation of cellular iron homeostasis or oral iron chelation might influence clinical disease.

## Supporting information

S1 Fig *B*. *pseudomallei* infection regulates transcription of HAMP and FPN in macrophages in a time-dependent manner. BMM were infected with *B*. *pseudomallei* at MOI 50 and harvested at the indicated time points. RNA was analysed for gene expression of divalent metal transporter-1 (Dmt1, n = 6), hepcidin antimicrobial peptide (HAMP, n = 4) and ferroportin (FPN, n = 4), and ferritin heavy chain 1 (FTH1, n = 5) by qRT-PCR. Data are expressed as mean with SEM (n = 4). Statistical analyses were done using Student’s *t-*test (*p<0.05, ***p<0.001).
(TIFF)

S2 Fig Iron loading stimulates HO-1 and FTH1 expression, intracellular iron content and *B*. *pseudomallei* growth. BMM were treated with ferrous sulphate (FeSO_4_, 50 μM) or corresponding vehicle (A. bidest) for 20 hours followed by infection with *B*. *pseudomallei* at MOI 50. **(A)** 24 hours after infection gene expression of HO-1, FPN, and FTH1 was analysed by qRT-PCR. Data are expressed as mean with SEM (n = 5). **(B)** Intracellular free iron levels were monitored after 24 hours using the iron-sensitive fluorescent probe Phen Green SK. Data are presented as mean with SEM of triplicate determinations (n = 3). (A, B) Comparison of groups was performed using one-way ANOVA and the Bonferroni post-hoc test (*p<0.05, **p<0.01, ***p<0.001). **(C)** BMM or murine hepatoma Hepa1-6 cells were treated with FeSO_4_ (50 μM) or corresponding vehicle for 20 hours followed by infection with *B*. *pseudomallei* at MOI 50 (BMM) or MOI 200 (Hepa1-6). Invasion (0 h) and intracellular growth (6 h, 24 h) of *B*. *pseudomallei* were examined by kanamycin protection assay. Data are presented as mean with SEM of triplicate determinations (n = 3). **(D)** LB broth or M9 minimal medium with or without FeSO_4_ (50 μM) or corresponding vehicle was inoculated with *B*. *pseudomallei*. The optical density (OD) at 650 nm and colony forming units (CFU)/ml were determined at indicated time points. Data are presented as mean and SEM of duplicates. (C, D) Statistical analyses were conducted using Student’s *t-*test (*p<0.05, **p<0.01, ***p<0.001).
(TIFF)

S3 Fig Immune defense functions are not affected by iron chelating drug in *B*. *pseudomallei* infected macrophages. BMM were treated with DFO (50 μM) or corresponding vehicle (A. bidest) for 20 hours followed by infection with *B*. *pseudomallei* at MOI 50. **(A)** 24 hours after infection gene expression of Ncf1 (n = 6), iNOS (n = 5), TNFα (n = 6), IL-6 (n = 6), IL-1β (n = 4), Nlrp3 (n = 6), and Lcn2 (n = 6) was analysed by qRT-PCR. **(B)** Lcn2 secretion in supernatants of DFO (100 μM) -or vehicle-treated BMM was measured by ELISA (n = 3). (A, B) Data are expressed as mean with SEM. Comparison of groups was done using one-way ANOVA and the Bonferroni post-hoc test (*p<0.05, **p<0.01, ***p<0.001). **(C)** Cleavage of caspase-1 and -7 and expression GAPDH were detected by immunoblot in cell lysates of DFO- or vehicle-treated BMM at 24 hours after infection with *B*. *pseudomallei*. **(D)** Cytotoxicity was determined as lactate dehydrogenase (LDH) release in cell supernatants of DFO- or vehicle-treated *B*. *pseudomallei*-infected BMM. Data are presented as mean with SEM of triplicate determinations (n = 3). Statistical analyses were conducted using Student’s *t-*test (***p<0.001).
(TIFF)

S4 Fig Iron homeostasis is modulated during murine melioidosis following iron supplementation or chelation. FAC (5 mg/kg), DFO (100 mg/kg) or vehicle (D-PBS)-treated C57BL/6 mice were intranasally inoculated with *B*. *pseudomallei* at 500 CFU and sacrificed 48 hours after infection. **(A)** Gene expression of FPN, HAMP, and FTH1 in the liver was analysed by qRT-PCR. Data are presented as mean with SEM (n = 8). The relative expression of target genes was normalized to the mean of the control, which was set to one. Total iron concentration **(B)** in the liver (n = 8) and **(C)** in serum (n = 10) and **(D)** release of hepcidin in serum (n = 18) were determined 48 hours after infection. Data are expressed as mean with SEM. Statistical analyses were done using Student’s *t-*test (*p<0.05, **p<0.01).
(TIFF)
